# Memoirs of the Royal Medical Society at Paris, Vol. II

**Published:** 1783

**Authors:** 


### IV. *Memoirs of the Royal Medical Society at Paris, vol.* II. *(continued from page* 244.)

ANATOMY.—1. *A caſe of Carcinomatous ulcer of the heart. By* M. Carcaſſone, *phyſician at Perpignan.*—A girl, 22 years old, came into the hoſpital at Perpignan with chancres, condylomata, and other ſymptoms of the lues venerea. She complained alſo of a ſenſe of weight in the left ſide of her thorax between the fifth and ſixth ribs.—The venereal ſymptoms yielded to proper remedies, but the ſenſation in her cheſt became gradually more and more troubleſome, and at the end of five or ſix months, was attended with lancinating pains. Her appetite at firſt was voracious, but ſhe ſoon voided her food by ſtool half digeſted. Her pulſe was, in general, ſmall and quick, and ſometimes when the pain in her breaſt was acute, her pulſe would intermit. As the diſeaſe advanced ſhe was occaſionally ſubject to ſyncope, and, by degrees, becoming incapable of lying down, ſhe was obliged to ſit in her bed leaning a little either backwards or forwards on her left ſide. The pain became gradually more intenſe, and the faintings more frequent, till the patient's death, which which took place about two years from the commencement of the difeafe. For about a year before her death inftead of hunger fhe had an averfion to food. On diffection three quarts of ferum were found in the left cavity of the thorax. The pericardium was almoft entirely deftroyed by a carcinomatous ulcer, which had eroded a great part of the fubftance of the heart. This ulcer was feparated from the right ventricle only by a few mufcular fibres which gave way to a flight preffure with a finger. The right auricle was enlarged and fchirrous, but the left was nearly in a natural ftate. The coronary veffels were in a varicous ftate. The fubftance of the heart was of a cartilaginous confiftence, and fo much enlarged as to meafure nearly twelve inches round below the auricles. The ulcer itfelf was upwards of nine inches in circumference. —2. *Anatomical obfervations. By* M. Vicq d'Azyr. —In this paper the author firft fpeaks of the mucous glands of the gall bladder. Thefe, he obferves, are more diftinct in quadrupeds than in the human fubject; and though moft numerous near the neck of the cyft, are fpread over its whole inner furface. His defcriptions are illuftrated by engravings. He next treats of the *membrana pupillaris* of the fœtus.—Wachendorf, dorf, who publiſhed a deſcription of this membrane in 1740, thought it was conſtantly of a brown or black colour. Two years afterwards Haller denied this. Albinus, who claimed the diſcovery, gave two engravings of this membrane. In one of theſe he merely marks its ſituation; in the other he delineates its veſſels, but in too ſmall a way, and in a manner not ſufficiently ſatisfactory. This has induced M. Vicq d'Azyr to give a more exact figure of it. The paper concludes with an anatomical problem, which the author deems intereſting to the theory of luxations. Does the Ulna move in pronation and ſupination, and if it does, what is its motion? This is the queſtion which he has undertaken to ſolve. He is convinced by experiments, that while the pronator quadratus and radialis externus roll the radius inwards and downwards, the extenſors of the Ulna contract and move its lower extremity, or that next the wriſt, a little backwards, by which means the pronation is facilitated and rendered more complete. In ſupination, on the contrary, the brachialis ſuperior, he obſerves, bends the Ulna a little, and aſſiſts in this motion of the fore arm by raiſing the lower extremity of that bone.—3. *Deſcription of two maſſes of hair found*

*in*

*in the ſtomach and bowels of a boy ſixteen years old.* *By* M. Baudamont, *ſurgeon at Verdun.*—The patient, who is the ſubject of this hiſtory, had from his infancy delighted in ſwallowing hairs, ſo that he not only plucked his own but likewiſe thoſe of his brother and others. This ſingular taſte increaſed as he grew up. The hairs thus ſwallowed formed an oval mat, which might be felt a little below the pit of his ſtomach. At length his ſtomach became painful, a fever ſucceeded, and terminated in death. On diſſection two maſſes of hair were diſcovered. The largeſt of theſe completely filled the ſtomach, and extended almoſt throughout the duodenum. This had a cherry-ſtone for its nucleus. The other ſmaller maſs was in the jejunum. The two, when recent, were moiſtened with a very fœtid mucus, and weighed two pounds and an ounce. In a dried ſtate their weight was reduced to eleven ounces and a half.

*Medical Chemiſtry.*—1. *Of the preparation of Lunar Cauſtic.* *By* M. Bucquet.—2. *Of the nature of the eſſential ſalt of Tamarinds.* *By* M. De Laſſone, jun. M.D. Theſe experiments prove this ſalt to be of the ſame nature as Cream of

Tartar.—3. *Remarks on potable waters.* *By* M. Thouvenel.—4. *Account of a bone which in calcination became of a pale green colour, from being in contact with ashes of oak loaded with fixed alkali.* *By* M. le Comte. The author contents himself with relating this fact, without attempting to explain it.—5. *A solution of mercury in fixed air, recommended as a remedy, by* M. Nicolas.—The mode of preparing this remedy is not described.

*Botany and the natural history of drugs.*—1. *On Colombo Root.* *By* M. Bertrand de la Gresie.—These remarks contain nothing new to the English reader.—2. *On the virtues of Muscus Pyxioides in the chin-cough.* *By* M. Van Woensel, M. D. *formerly in the Russian service.*——This virtue has been noticed by Gerard, Willis, Ray, and other writers. The author of the present paper, when physician to the corps of Noble Cadets at Petersburg, experienced its good effects in a great number of cases, given in decoction sweetened with syrup.—3. *A description of the flower and fruit of the Clove-tree.* *By* M. Ceré, *director of the king's garden in the Isle de France.* This plant, which it seems has been erroneously described by Rumphius, belongs to the 13th class (*Polyandr. Monogyn.*) of Linnæus.—4. *On the use of Ivy in bread.* *By* M. de la Maziere,

M. D.

M. D. *physician at Poictiers.*—A case is related in which bread made of the leaves of ivy brought on a fatal colic.—5. *On the use of the Lactuca Virosa. By* M. Durande, M. D. *at Dijon.* This physician, who has tried this remedy in several cases, observes that it does not deserve the praise bestowed on it by Dr. Collin in the dropsy, but he has found it useful as a sedative.—6. *On the effect of tobacco applied externally.*—In the Edinburgh essays, (vol. 2.) we read of tobacco being beat up with vinegar, applied to the stomach and exciting vomiting. We have here another case of the same kind.—7. *On a Syrup made with the bark of the root of Simarouba. By* M. Badier, *of Guadaloupe.*—This syrup is recommended for the dysentery.——8. *Account of the effects of smutty rye. By* M. le Brun.——A wet season in the province of Auch produced a great deal of smutty rye, and a variety of bad effects among the poor inhabitants who made it into bread. A dog fed with it soon lost his hair, became emaciated, and could hardly walk; and ducks and fowls to whom it was given were deprived of their feathers.—9. *Of the effects of the leaves of the Palma Christi applied to the head. By* M. Maziere, M. D.— The case of a woman is related who applied these leaves for a pain in

 her her head. They are ſaid to have occaſioned a blindneſs, which was relieved by bliſters.—— 10. *Of the ill effects of muſhrooms.*—Several inſtances of their pernicious effects are related, but in all of them relief was obtained by emetics.

*Medical Philoſophy.*—1. *An account of a voyage to the Levant, with remarks on the diſeaſes that prevail there, the nature of the ſoil, and the temperament of the inhabitants.*—The ſociety having been informed in 1777, that Baron de Tott was about to travel into Egypt, preſented a paper to him containing ſeveral queries, which he undertook to ſhew to thoſe perſons who might be the moſt likely to give ſatisfactory anſwers to them. M. Hollande, a phyſician, who accompanied the baron in his tour, and M. Mallet de la Broſſiere, a phyſician, reſident in the iſland of Juda, are the perſons who have afforded the ſociety the beſt information relative to the objects of their inquiry. The obſervations of theſe gentlemen tend to prove that the plague is not connected with any particular ſtate of the air; that no epidemic prevails amongſt other animals during the time of the plague, and that they are not affected by the virus of this diſeaſe; that it is wrong to conſider Egypt as the the ſource of this contagion, it being certain that for the ſpace of ten years it has not prevailed in that country, although within that time it has repeatedly appeared at Conſtantinople; that it is not brought by the winds from the interior parts of Africa into Egypt; and that the Franks, by avoiding communication with the infected, eſcape the diſeaſe. We are told, that at Smyrna the plague was in a houſe adjoining to the European diſtrict, but without affecting the latter, till a young Engliſh Lady walking on the terraſs of her houſe, which was lower than that of the infected habitation, received the contagion from the emptying of a bed-pan on the adjoining terraſs, and died the next day with evident ſymptoms of the plague.

The ceſſation of the plague is not attended with any remarkable change in the ſtate of the air. It has only been obſerved, in general, that the violent heats of ſummer, and the ſcorching blaſt of the ſouth wind ſuſpend or put a ſtop to it, as it were by enchantment; and that when it prevails in Egypt, it is weakened and gradually extinguiſhed by the ſummer ſolſtice.

It is not true, we are told, that after the retreat of the Nile the earth is covered with animals, mals, the corruption of which infects the air. This river in retiring leaves no fish or other animals on the surface of the earth; no smell is perceptible in the air, and the superabundant moisture is soon exhaled by the sun. There are but few places, it is added, where there are stagnant waters in Egypt, because the whole country is intersected with canals in which they run off, and when these are left dry, the bottom, being sandy, dries without exhaling any bad smell.—2. *Observations on Natrum, the cultivation of rice, and a disease which prevails among the inhabitants of Aleppo. By* M. Hollande, M.D. —The natrum in Egypt is principally got from three lakes. The disease which prevails among the inhabitants of Aleppo, and which commonly attacks them during infancy, begins with a pimple in the face, which spreads without pain and terminates very slowly without any remedy. Its duration is from six to fifteen months. Topical applications have been laid aside because they have been found to irritate and prolong the disease. A cicatrix remains at the place where the pimple began. The middle of the cheek is the part most commonly attacked, but the disease sometimes appears in other parts of the body. A similar complaint prevails at Bassora, and and along the whole weftern fhore of the Perfian Gulph.—3. *On the bite of the Scorpion. By* M. Mallet de la Broffiere, M. D.—An account is given of two cafes at Tunis, in which volatile alkali adminiftered internally proved fuccefsful. —4. *An account of a fever which prevailed in the iflands fituated along the coaft of Zanguebar. By* M. de la Peyre.——5. *Report of a committee appointed to inquire into the effects of a manufactory of antimonial preparations.*—Perfons living in the neighbourhood of this manufactory had complained of it as a nuifance. The fociety were applied to by the attorney general to determine whether it ought to be confidered as injurious to the public. The committee (Meffieurs Macquer, Mauduyt, and Bucquet) offer feveral reafons to prove that the health of the neighbourhood cannot be affected by it.—6. *Remarkable effects of lightning. By* M. Dantic, M. D.—7. *Refult of experiments to afcertain the refpective dilatation and condenfation of mercury and fpirit of wine in thermometers. By* Father Cotte. —Thefe experiments are intended to prove that for thermometrical purpofes mercury is preferable to fpirit of wine, but from the facts lately eftablifhed at Hudfon's Bay relative to the freezing ing of mercury †, it appears that in cold countries ſpirit thermometers claim the preference.

We come now to the MEMOIRS or ſecond part of the volume, which conſiſts of ſuch papers as the ſociety have thought fit to publiſh entire. Here, as in the hiſtorical part, we ſhall give the title of every paper, and add ſome account of ſuch as admit of abridgment, or appear to be the moſt intereſting.

1. *Conſtitution of the year* 1777 *at Paris. By* M. Lorry.—2. *Conſtitutions of the years* 1777 *and* 1778. *By* M. Geoffroy.—3. *Reflections on the periodical head-ache obſerved at Paris at the latter end of April and beginning of May* 1778. *By* M. Coquerau.—4. *An eſſay on the Suette which prevailed at Hardivilliers in Picardy in May* 1773. *By Abbé* Teſſier.—The *Suette* § of Picardy is an epidemic fever, one of the principal ſymptoms of which is a profuſe ſweating, particularly on the breaſt, and accompanied frequently with miliary eruption. By adopting a cooling regimen, freſh air, and venæſection at the beginning, this diſeaſe ſeldom proved fatal, though before this plan was purſued great numbers had fallen martyrs to a heating regimen.—5. *An eſſay*

† See p. 205. § See p. 54.

*on*

*on a petechial and contagious fever which for several years has prevailed at Josselin in Brittany, and in the adjacent parishes.* *By* M. Robin de Keriavalle.—6. *An essay on the medical topography of Marseilles and its neighbourhood.* *By* M. Raymond, M. D.—This is a very elaborate and judicious dissertation. It fills 74 pages of the volume, and contains many curious facts.—From the author's inquiries it appears that at Marseilles the number of inhabitants amounts to 68,508. The proportion of male inhabitants is greater than that of the females, which is contrary to what is observed in the other towns of Provence. The number of boys under 12 years is to that of the girls as 33 to 32, which is nearly in the same proportion as the births. The number of children under 12 years old is to that of the rest of the inhabitants as 1 to 4, 2; which is much less than in other parts of the province, where it is as 1 to 3, 2, and which shews that there is a greater proportion of unmarried persons in Marseilles, that the marriages are less fruitful, and the mortality of children greater.

From the year 1750 to 1764 inclusive, the average number of births, deaths, and marriages has been as follows:

| | |
|---|---|
| Births | 3352 |
| Deaths | 3157 |
| Marriages | 673 |

The number of foundlings to that of legitimate children during the ſame ſpace of time has been in the proportion of 1 to 8. From the tables of births it appears, that the women conceive moſt in autumn, next to that in ſummer, and leaſt of all in winter. October is found to be the month remarkable for the greateſt, as March is for the leaſt number of conceptions.—The mortality is greateſt in the two firſt years of life, and from the 60th year to the 70th, and it is leaſt between the ages of 20 and 30. The mortality during the firſt year of life is in the proportion of 1 to 39, and during the firſt five years is found to be equal to $\frac{1}{8}$ of the total of deaths; ſo that of 18 children 10 die before they are ſix years old.—The chance of life at Marſeilles is calculated to be 25, 4 years. —The Hotel Dieu at Marſeilles admits on an average 4058 patients every year. Of this number 1 in 7 die. This great mortality is aſcribed to the want of freſh air, and to the wards being too much crowded, two or more patients being often placed in the ſame bed.—The Charity Hoſ-

Hoſpital is an extenſive building, for the reception of children, who are admitted into it at the age of 7, and kept there till they are 17 or 18. In the ſpace of 20 years, viz. from 1754 to 1773, 10,454 have been admitted into it, of whom have died 1084. One of the cauſes of this mortality is ſaid to be the bad quality of the bread.——The hoſpital for foundlings and orphans is in an open ſituation at the weſt ſide of the city. In a period of 20 years 1750 children have been placed in it, of which number 293 have died.

Speaking of the endemial diſeaſes that prevail at Marſeilles, M. Raymond obſerves, that the acute diſeaſes are to the chronic in the proportion of 9 to 5. Intermittents are ſaid to be rare, and eaſy of cure. Their frequency, when compared with that of continued fevers, is rated in the proportion of 3 to 22. This is aſcribed to the dryneſs of the ſoil and atmoſphere. The fluor albus, we are told, is common, and occurs even in children five years old.——7. *Obſervations on the diſeaſes of Champſaur in Dauphiny. By* M. Villar, M. D.——8. *Remarks on the operation of certain remedies, and particularly of opium. By* M. Lorry.—Whytt has aſſerted that the action of chalybeates is confined to the primæ viæ. In proof of this he quotes an experiment related by the late Dr. Wright (*Philof. Tranf.* 1750) who examined the chyle of a dog that had been fed with a mixture of bread and fal martis, and could difcover in it no marks of iron. But in the paper before us M. Lorry afferts, that the urine of a patient who had drank the chalybeate water of Paffy in great quantity for 40 days, began at the end of that time, though not before, to exhibit marks of iron when tried with a tincture of galls. This impregnation, he adds, continued many days after the patient had defifted from the ufe of the water.—Some facts are related to confirm the opinion that the narcotic power of opium refides in its volatile part.——9. *Effects of Electricity in eighty-two patients.* *By* M. Mauduyt.——10. *Remarks on the general effects, nature, and ufe of the electric fluid confidered as a remedy.* *By* the Same.—11. *Supplement to an effay on the Hydrophobia* *. *By* M. Andry.—12. *On the management of fheep.* *By* M. Daubenton.—13. *On the lateral operation as defcribed by Chefelden, and on the means of rendering the practice of it more eafy.* *By* M. Vicq d'Azyr.—This ingenious writer is of

* See vol. III. p. 288.

opinion opinion that the staff recommended by Chefelden has too small a curve, so that when the point of the knife has reached it, the operator is obliged to lower his wrist in order to carry his instrument through the neck of the bladder. To avoid this inconvenience he recommends a staff with a larger curve. The introduction of it, he observes, will be a little more difficult, but it will be more useful when introduced. He likewise remarks that the knife employed by Chefelden is not of a sufficient length for adults when they are fat.—14. *On the effects of smutty rye. By* Abbé Tessier. --The author fed several ducks and pigs with this substance. In all these animals it brought on gangrene and death. His experiments prove that animals are averse to it. —15. *Observations on the disease among the horned cattle in Flanders in* 1774. *By* M. Berg, *amman of Brussels.*—This essay obtained the premium offered by the society in 1776.

---

V. Jani

